# Collection and use of EQ-5D for follow-up, decision-making, and quality improvement in health care - the case of the Swedish National Quality Registries

**DOI:** 10.1186/s41687-020-00231-8

**Published:** 2020-09-16

**Authors:** Olivia Ernstsson, Mathieu F. Janssen, Emelie Heintz

**Affiliations:** 1 Department of Learning, Informatics, Management and Ethics (LIME), Karolinska Institutet, Tomtebodavägen 18A, SE-171 77 Stockholm, Sweden; 2 Section Medical Psychology and Psychotherapy, Department of Psychiatry, Erasmus MC, Dr. Molewaterplein 40, Rotterdam, 3015 GD the Netherlands; 3 EuroQol Research Foundation, Rotterdam, the Netherlands

**Keywords:** EQ-5D, Patient-reported outcome measures, PROM, Health-related quality of life, Quality registries, Quality improvement, Routine measurement

## Abstract

**Background:**

The Swedish National Quality Registries (NQRs) contain individual-level health care data for specific patient populations, or patients receiving specific interventions. Approximately 90% of the 105 Swedish NQRs include any patient-reported outcome measure, with EQ-5D being the most common. As there has been no general overview of EQ-5D data within the NQRs, this study fills a knowledge gap by reporting how the data are collected, presented, and used at different levels of the Swedish health care system.

**Methods:**

All 46 NQRs with a license for the use of EQ-5D were included. Information was retrieved from the registries’ annual reports or from websites, using a template that was subsequently sent to each registry for completion and confirmation. If considered necessary, the contact was followed-up with an interview, either in-person or over the telephone. The uses of EQ-5D were categorised as denoting usage for follow-up, decision-making, or quality improvement in Swedish health care.

**Results:**

In total, 41 of the 46 licensed registries reported collection of EQ-5D data. EQ-5D is most commonly collected within registries related to the musculoskeletal system, but it has a wide application also in other disease areas. Thirty-six registries provide EQ-5D results to patients, clinicians, or other decision-makers. Twenty-two of the registries reported that EQ-5D data are being used for follow-up, decision-making or quality improvement. The registries most commonly reported use of data for assessing interventions, and in quality indicators to follow-up the quality of care at a national level.

**Conclusion:**

Collection and use of EQ-5D data vary across the Swedish NQRs, which may partly be accounted for by the different purposes of the registries. The provided examples of use illustrate how EQ-5D data can inform decisions at different levels of the health care system. However, there is potential for improving the use of EQ-5D data.

## Background

There are 105 National Quality Registries (NQRs) in Sweden [1]. The registries contain structured health care data for specific patient populations, or patients receiving specific interventions. The data are collected and registered nationally by routine health care practice providers, for the purpose of monitoring and developing the quality of Swedish health care [2]. Many of the registries began as an initiative of one or several local health care professionals, either at a specific hospital or at a group of hospitals (the first one was in 1975) [2]. As years went by, more and more registries were launched, and eventually, the need for a national support organisation became evident. Registries now strive for a high affiliation of clinics, as well as high and complete coverage of eligible patients.

The Swedish Government has supported the registries financially since 1990, and the registries are today jointly financed by the Swedish Government and the 21 health care regions that have the main responsibility for providing health care to their inhabitants in the highly decentralised Swedish health care system [2]. In order to receive financial support, the registries are required to take part in an annual monitoring process, and the financial support has to be approved by an Executive Committee [1]. Each registry is supported by an inter-professional team of health care professionals and researchers, with patient representatives also often included [2]. However, the registries have different purposes and needs, which influence their organisation and structure.

From their commencement, the registries have focused on clinical measures such as survival, complications, and other clinical signs of disease progression in the follow-up of health care [2]. However, it has become more common to include one or more patient-reported outcome measures (PROMs). PROMs are standardised self-reported measures that are typically developed to capture a person’s perspective on outcomes related to their health status or health-related quality of life (HRQoL), such as symptoms, functioning, overall health, or well-being [3].

Several potential uses of PROMs in clinical practice have been identified [4–8]. At an individual level, PROMs may, for example, be used as a screening tool (e.g. to detect depression or anxiety), to monitor effects of a patient’s treatment, and/or to get patients more involved in decisions regarding their own care (shared decision-making) [4, 6]. In addition, results on an aggregated level may be used in decision aids, to provide patients and health care professionals with information regarding the impact of different treatment options, to inform health technology assessments or reimbursement decisions regarding the effectiveness of health care interventions, to evaluate the quality of care within a practice, and/or to enable providers to benchmark their performance with others [5, 7, 8].

In a previous review, it was reported that almost 90% of the NQRs have included at least one PROM [9]. The most commonly collected PROM was EQ-5D. EQ-5D is a measure of health, and consists of two parts: a questionnaire with five items (the descriptive system) that can be summarised in a health profile; and a visual analogue scale (EQ VAS) that patients may use to rate their own health on a scale between 0 (worst imaginable health) and 100 (best imaginable health) [10, 11]. The five items in the descriptive system each represent a dimension of a person’s health status; mobility, self-care, usual activities, pain/discomfort, and anxiety/depression. There are currently two different versions for adults (EQ-5D-3L and EQ-5D-5L), and a child-friendly version for children and adolescents (EQ-5D-Y). The difference between the two versions for adults is mainly the number of response levels on each question (three or five), but the questions have also been slightly modified in the newest version (EQ-5D-5L) [12]. The responses to the five questions may be converted to an index value that can be used for calculating quality-adjusted life years (QALYs), which is the recommended health outcome measure used in cost-effectiveness analyses of health care interventions in many countries [13, 14]. The index values are obtained from existing value sets that are typically developed for reimbursement purposes in each specific country. EQ-5D value sets have been derived and published for many countries worldwide [15].

The implementation and use of PROMs in clinical practice is associated with several challenges [16–21]. These challenges include: the patients’ willingness to respond to topics addressed in different PROMs; choosing the most adequate PROM; interpreting results; skepticism regarding their usefulness and associated workload; and how the measurements fit into the way the care is organised, including (IT) infrastructure. More generally, low interpretability of output data (e.g. by patients and care givers), as well as survey fatigue, have been identified as barriers to the use of clinical registry data in quality improvement, research, and interactions with patients [22]. Altogether, these findings highlight the importance of not only focusing on the data collection when exploring the implementation of PROMs in routine health care, but also the need to study the actual use of PROMs, and their implications in terms of improvement of patient care and/or health outcomes.

Experiences from some previous large-scale initiatives for implementing PROMs in routine care settings have been described in the literature [21, 23]. Nevertheless, reports of real-world implementation initiatives are still limited [5, 24, 25]. Studying the collection and use of EQ-5D within the Swedish NQRs represents a unique opportunity to learn from a real-world case of a large-scale application of a specific PROM in routine health care. There has so far been no overview available on how these registries collect and use the EQ-5D data. This study fills the gap by reporting how EQ-5D data are collected, presented, and used at different levels of the Swedish health care system. The results can be useful for clinicians, researchers, and decision-makers at different levels of the health care system in Sweden, and in other countries, who may learn from the experiences and progress of the Swedish national quality registries.

## Methods

The aim of the study was to increase knowledge on how EQ-5D data are collected within the Swedish NQRs, and how the data are made available and are being used in the Swedish health care system. All 46 registries with a license to use EQ-5D at the point of data collection were included. Ten of these NQRs were sub-registries within the Swedish Neuro Registries.

Information concerning each registry was based on information available online, as well as on personal communications with representatives from all 46 registries. The representatives were persons engaged in the management and/or development of the registry, for example as registry holder or being involved in the registry’s work with PROMs and/or PREMs.

Prior to the data collection, a template including questions regarding the registries’ collection and use of EQ-5D was developed by the authors (Table S1). The questions concerned characteristics of the registry in general, the collection of PROM data, and specifically EQ-5D data, administration of the PROM instruments, and use of PROM data for quality improvement and decision-making. The template was completed with information from the registries’ annual reports and/or websites, and sent by e-mail to a representative from each registry for confirmation and completion. If considered necessary, the e-mail contact was followed up with additional questions over e-mail, or in an interview, either in-person or over the telephone. The information was retrieved between August 2018 and June 2019.

For each reported use of the data, the representatives were asked to provide examples of how EQ-5D data from their registry are being used for that purpose. Subsequently, the resulting comprehensive information was condensed and tabulated. The contact persons for each registry were given the opportunity to confirm the information regarding how EQ-5D data are collected (Table S2–S12), presented, and used (Table 2).

The registries were categorised into disease categories (e.g., cancer, circulatory system), based on a classification system developed by the Swedish Association of Local Authorities and Regions (SALAR) [1] (Table 1). The registries were also categorised depending on their purpose. A “diagnosis registry” was defined as a registry that follows patients based on a diagnosis, and an “intervention registry” as a registry that follows patients based on them having received a specific intervention. This categorisation was performed by the authors in dialogue with colleagues at the Quality Registry Centre (QRC) Stockholm, based on the collected information regarding the criteria for including patients in each registry.

**Table 1:** Swedish National Quality Registries collecting EQ-5D data, 2018

Registry	Registry category	Registry type
National Prostate Cancer Register (NPCR) of Sweden	Cancer	Diagnosis
National Quality Registry for Head and Neck Cancer (SweHNCR)	Cancer	Diagnosis
National Quality Registry for Oesophageal and Stomach Cancer (NREV)	Cancer	Diagnosis
National Quality Registry for Atrial Fibrillation and Anticoagulation (AuriculA)	Circulatory system	Diagnosis
National Quality Registry for Congenital Heart Disease (SWEDCON)	Circulatory system	Diagnosis
National Quality Registry for Enhancement and Development of Evidence-Based Care in Heart Disease (Swedeheart)	Circulatory system	Diagnosis
The Swedish Catheter Ablation Registry	Circulatory system	Intervention
The Swedish Heart Failure Registry (SwedeHF)	Circulatory system	Diagnosis
The Swedish National Quality Registry for Ulcer Treatment (RiksSår)	Circulatory system	Diagnosis
The Swedish Register for Cardiopulmonary Resuscitation (SRCR)	Circulatory system	Intervention
National Quality Registry for Pituitary Disease	Endocrine organs	Diagnosis
National Quality Registry for Infectious Diseases	Infection	Diagnosis
National Quality Registry for Primary Immunodeficiency (PIDcare)	Infection	Diagnosis
Better management of patients with OsteoArthritis (BOA)	Musculoskeletal system	Intervention
National Quality Registry for Hip Fracture Patient Care (RIKSHÖFT)	Musculoskeletal system	Diagnosis
The National Quality Registry for Podiatric Surgery (RiksFot)	Musculoskeletal system	Intervention
The Swedish Ankle Registry (Swedankle)	Musculoskeletal system	Intervention
The Swedish amputation and prosthesis register (SwedeAmp)	Musculoskeletal system	Intervention
The Swedish Elbow Arthroplasty Register and The Swedish Shoulder Arthroplasty Register	Musculoskeletal system	Intervention
The Swedish Fracture Register (SFR)	Musculoskeletal system	Diagnosis
The Swedish Hip Arthroplasty Register	Musculoskeletal system	Intervention
The Swedish Knee Arthroplasty Register	Musculoskeletal system	Intervention
The Swedish Rheumatology Quality Register (SRQ)	Musculoskeletal system	Diagnosis
The Swedish Spine Register (SWESPINE)	Musculoskeletal system	Intervention
The National MMC Follow-Up Program and Quality of Care Registry (MMCUP)	Nervous system	Diagnosis
The National Quality Registry for Rehabilitation Medicine (Webrehab Sweden)	Nervous system	Intervention
The Swedish Neuro Registries – Hydrocephalus	Nervous system	Diagnosis
The Swedish Neuro Registries – Motor Neuron Disease	Nervous system	Diagnosis
The Swedish Neuro Registries – Multiple Sclerosis	Nervous system	Diagnosis
The Swedish Neuro Registries – Myasthenia Gravis	Nervous system	Diagnosis
The Swedish Neuro Registries – Narcolepsy	Nervous system	Diagnosis
The Swedish Neuro Registries – Parkinson’s Disease	Nervous system	Diagnosis
The Swedish Quality Registry for Pain Rehabilitation (SQRP)	Nervous system	Intervention
National Quality Registry for Child and Adolescent Habilitation, HabQ (HabQ CP, HabQ autism, HabQ Parental support)	Paediatrics	Intervention
National Quality Registry for Follow-up of Persons with Cerebral Palsy (CPUP)	Paediatrics	Diagnosis
BipoläR – the Swedish National Quality Register for Bipolar Disorder	Psychiatry	Diagnosis
National Quality Registry for Dependency (SBR)	Psychiatry	Intervention
The Swedish National Quality Register for ECT	Psychiatry	Intervention
SWIBREG – Swedish Inflammatory Bowel Disease Registry	Stomach and intestines	Diagnosis
National Quality Registry for Haemophilia	Other areas (rare diseases)	Diagnosis
National Quality Registry for Systemic Psoriasis Treatment (PsoReg)	Other areas (skin disease)	Diagnosis

The collected information regarding how EQ-5D data are made available was categorised to describe the different ways the results are provided to care givers, patients, and other decision-makers. The developed categories included feedback on an aggregated level, as well as on an individual level. Feedback on the aggregated level was categorised into feedback presented in: annual reports/websites; research publications; reports adapted for individual clinics, units, or teams; reports with patients as the target group; and other reports. Feedback on the individual level was categorised into: feedback directed to healthcare professionals entering data; and feedback to patients.

The use of the data was also categorised to describe how, and to what extent, the EQ-5D data are being used for follow-up, quality improvement, or decision-making at the individual level. The categories represent use in assessment of interventions, health economic studies, quality indicators, benchmarking, quality improvement, and in individual patient consultations. The categorisation was based on usage as defined by the representatives of the registries, but was modified by the authors in collaboration with the registries, using the information in the provided examples. For example, to be categorised as use in a quality indicator, the EQ-5D data from the registry had to be presented in a format that the registry or some other stakeholder defined as a quality indicator. In addition, we also required that the measure fulfilled some basic criteria in order to be defined as a quality indicator, i.e. that it represented a quantitative summary of health care data, had a clear direction of what indicates good or poor quality, and was relevant and important for improvement [2, 26]. The quality indicators could be based on outcome measures describing the health outcome of the patients (e.g. the patient’s responses to EQ-5D), or on process measures describing measurement and follow-up (e.g. the proportion of patients that are being followed-up with EQ-5D) [2].

To be defined as use in benchmarking, the usage reported by the registries had to represent a comparison of performance in terms of EQ-5D results between different health care providers, with the purpose of assessing the performance of each provider, and identifying needs for improvement [27]. These comparisons should preferably be case-mix adjusted, which means that the results have been adjusted for differences in the characteristics of the patient populations.

For the use of EQ-5D to be categorised as use in quality improvement, it was required that the registry representatives reported active use of EQ-5D data in efforts to improve the quality of care.

As it was found too difficult to separate the use of EQ-5D data for screening, monitoring, in decision aids, and for shared decision-making, these were combined in one category of use at an individual level, in patient consultations. Decision aids were defined as the presentation and use of individual or aggregated EQ-5D results to support health care professionals and/or patients in decisions regarding the individual patient’s care. Shared decision-making refers to the use of EQ-5D results in the discussion between a health care professional and a patient, with the purpose of encouraging patients to express their preferences regarding different treatment options, and to make decisions regarding their care, together with the care giver, based on the best available evidence [28].

## Results

### Collection of EQ-5D data

**Fig. 1:**
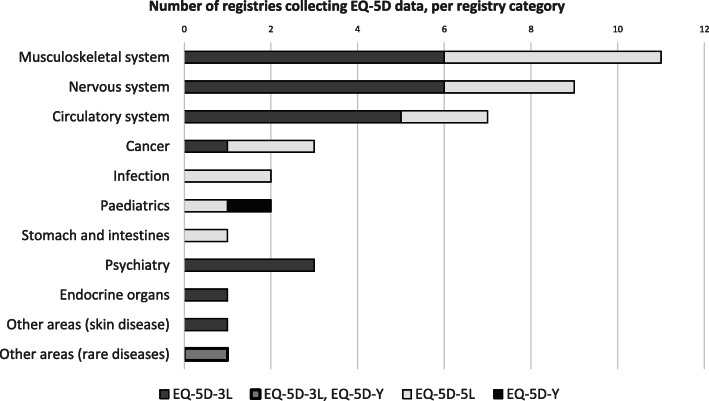
Number of registries collecting EQ-5D data (2018), per registry category

In total, EQ-5D data were collected in 41 of the 46 registries with a license for collection of EQ-5D (Table 1). EQ-5D data were most commonly collected in registries targeting conditions related to the musculoskeletal system, followed by those targeting conditions related to the nervous system, circulatory system, psychiatry, cancer, infections, paediatrics, obstetrics and gynaecology, stomach and intestines, endocrine organs, skin diseases, and rare diseases (see Table S2–S12 and Fig. 1). Fifteen of the registries with collection of EQ-5D data were intervention registries, and 26 were diagnosis registries (Table 1).

**Fig. 2:**
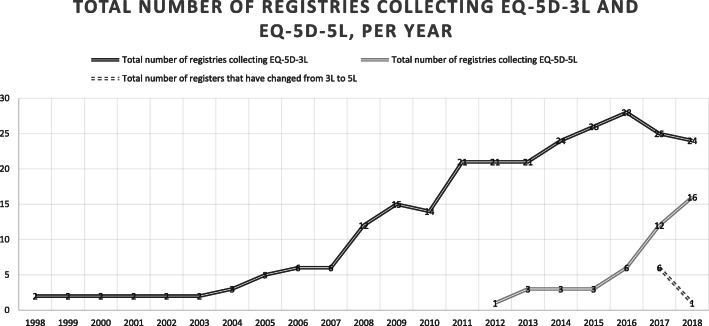
Number of registries collecting EQ-5D-3L and EQ-5D-5L, per year (1998–2018)

The two registries that first initiated collection of EQ-5D data started in 1998 (Fig. 2). These registries were the Swedish Spine Register (SWESPINE), and the National Quality Registry for Rehabilitation Medicine (Webrehab Sweden). Since 2004, when the Swedish Elbow Arthroplasty Register and The Swedish Shoulder Arthroplasty Register started collecting EQ-5D data, the number of registries collecting EQ-5D has gradually increased. The National Quality Registry for Respiratory Diseases intended to start collecting EQ-5D data for both adults and children with asthma and chronic obstructive lung disease, but had not begun to at the time of analysis for this study. In addition, four of the ten sub-registries within the Neuro registries did not collect EQ-5D data.

Twenty-three registries reported collection of EQ-5D-3L data, and 16 registries reported collection of EQ-5D-5L data. Further, one registry reported collection of EQ-5D-Y data, and one reported collection of both EQ-5D-3L and EQ-5D-Y data (Fig. 1, Table S2–S12). Among the registries collecting EQ-5D-5L data, seven registries had changed from EQ-5D-3L in 2017 (*n* = 6) or 2018 (*n* = 1). Five of these were registries within conditions related to the musculoskeletal system. All but four of the registries containing EQ-5D data have included EQ VAS since the start of EQ-5D collection. In one additional registry, the collection of EQ VAS started when changing to EQ-5D-5L in 2017.

The frequency of measurements varies between the registries. EQ-5D is either measured before and after interventions (*n* = 14), continuously at specific time points (*n* = 12), at baseline and at one or several follow-ups (*n* = 5), at one or several follow-ups without baseline (*n* = 3), once per patient (n = 3), at specific ages (n = 1), before injury (retrospectively) and at follow-up (*n* = 2), or whenever the patient wants to (n = 1). Among those measuring before and after intervention (n = 14), most registries reported having two or more follow-up measurements (*n* = 11). The registries with continuous measurements at specific time points (*n* = 12) were all diagnosis registries in which EQ-5D was measured every 6 months, annually, at each health care visit or health care contact, or every second or third year.

The most common mode of administration was to use paper questionnaires only (*n* = 20), followed by the use of either paper or web questionnaires (*n* = 10), web questionnaires only (*n* = 6), and either paper, web questionnaires, or interviews (n = 2). The remaining three registries reported collection of EQ-5D data using interviews (n = 1), paper questionnaires or interviews, (n = 1), or a paper questionnaire followed up with a telephone interview (n = 1).

The number of measurements ranged from approximately 60 individuals with EQ-5D measurements in the National Quality Registry for Atrial Fibrillation and Anticoagulation (AuriculA) (since year 2018), and in the Swedish Neuro Registry for Myasthenia Gravis (since year 2016), to more than 440,000 measurements in the Swedish Hip Arthroplasty Registry (since year 2008) (Table S2–S12).

### Presentation of EQ-5D data

The registries reported that EQ-5D results are provided to care givers, patients, and other decision-makers through several channels. Aggregated EQ-5D results are most commonly made available through the registries’ annual reports or websites (*n* = 29) (for examples of visualizations, see [29–31]). However, it is also relatively common for the clinics’ own aggregated EQ-5D data to be presented in reports directed to each clinic or unit, and/or as feedback to professional teams (*n* = 20). In addition, several registries reported that the EQ-5D results have been included in scientific publications (*n* = 18).

Some registries reported that data on an individual patient level are made available to health care professionals (*n* = 17), and/or patients (*n* = 12), and that data on an aggregated level are made available to patients (*n* = 5), and/or in other types of reports (*n* = 6). Other types of reports include national online tools for monitoring health care, public reports to governmental agencies, master theses, or other publications regarding the health status of the patient group. Only five registries reported that they are not communicating EQ-5D data through any channel. Of these, four registries began collecting EQ-5D in 2016 or 2018.

### Use of EQ-5D data for follow-up, quality improvement, and decision-making

Our study shows that the EQ-5D data from the registries are being used in quality indicators, for assessment and/or economic evaluations of interventions, for quality improvement, benchmarking, and/or in individual patient consultations (Table 2). The use of the data is described in more detail below. Representatives from 67% (10/15) of the intervention registries reported use of EQ-5D data (Table 2). The corresponding number for the diagnosis registries was 46% (12/26). Nevertheless, representatives from several registries reported that it is unclear if the data are being used for any purpose, or that it is not being used at all (*n* = 19). Four of these registries began collecting EQ-5D data in 2017 or 2018.

**Table 2:** Frequency of registries reporting use of EQ-5D data, per category of registry and category of use

	Reported use of EQ-5D data ^**a**^							
	Registries collecting EQ-5D data	Registries reporting use of EQ-5D data	Assessment of interventions	Health economic studies	Quality indicators	Benchmarking	Quality improvement	Use at individual level
	n	n	n (% of registries using EQ-5D)					
All registries	41	22	10 (45)	5 (23)	10 (45)	7 (32)	6 (27)	7 (32)
Reported use by type of registry								
	n	n	n (% of registries using EQ-5D, within type of registry)					
Diagnosis	26	12	3 (25)	3 (25)	4 (33)	2 (17)	3 (25)	5 (42)
Intervention	15	10	7 (70)	2 (20)	6 (60)	5 (50)	3 (30)	2 (20)
Reported use by registry category								
	n	n	n (% of registries using EQ-5D, within registry category)					
Musculoskeletal system	11	9	7 (78)	1 (11)	4 (44)	4 (44)	2 (22)	2 (22)
Neurological system	9	4	1 (25)	2 (50)	1 (25)	2 (50)	3 (75)	3 (75)
Circulatory system	7	3	1 (33)	1 (33)	2 (67)	0 (0)	0 (0)	0 (0)
Cancer	3	1	0 (0)	1 (100)	0 (0)	0 (0)	0 (0)	0 (0)
Psychiatry	3	1	0 (0)	0 (0)	1 (100)	0 (0)	0 (0)	0 (0)
Paediatrics	2	2	0 (0)	0 (0)	1 (50)	0 (0)	1 (50)	1 (50)
Other areas	2	2	1 (50)	0 (0)	1 (50)	1 (50)	0 (0)	1 (50)
Infection	2	0	–	–	–	–	–	–
Endocrine organs	1	0	–	–	–	–	–	–
Stomach and intestines	1	0	–	–	–	–	–	–

^**a**^Note that each registry can report several categories of use of EQ-5D data

#### Use of EQ-5D data in assessment of interventions and health economic studies

Ten registries reported that the collected EQ-5D data are being used to assess health care interventions. For example, EQ-5D data from the National Quality Registry for Atrial Fibrillation and Anticoagulation (AuriculA) have been used in a study that aimed to assess the effectiveness and cost-effectiveness of a risk score-based treatment of patients with atrial fibrillation [32]. Furthermore, EQ-5D data from other registries have been used to assess the outcome of an osteoarthritis learning programme [33], biological treatment in psoriasis [34], pain rehabilitation [35], treatment of fractures [36], hip arthroplasty [37], shoulder arthroplasty [38], spine surgery [39], knee arthroplasty [30], foot surgery [40], and total ankle replacement [41, 42]. In addition to AuriculA, four registries (SQRP, Swespine, MMCUP, and NREV) described ongoing collaborations with health economists to investigate the health economic aspects of the disease, or specific interventions.

#### Use of EQ-5D data in quality indicators

In total, ten registries reported use of EQ-5D data in quality indicators (Table 3). The majority of the described quality indicators are based on the outcome results of EQ-5D, but there are also three examples utilising the number of EQ-5D measurements as a process-based quality indicator for assessment of the follow-up process. The quality indicators (either outcome- or process-based) are presented in the registries’ reports, at national platforms, or on online tools, such as “Health care in numbers” (“Vården i siffror”), or the SVEUS platform. “Health care in numbers” is a national online tool for quality improvement in Swedish health care. It is administrated by SALAR and financed by the 21 regions that are responsible for providing health care in Sweden [50]. SVEUS is a platform for real time follow-up of health care data, and it is a collaborative initiative between SALAR and the participating health care regions [45].

**Table 3:** Use of EQ-5D-data in quality indicators

Registry	Description of quality indicator
**Quality indicators based on outcome measures - published on national platforms**	
The registry for Better management of patients with OsteoArthritis (BOA)	EQ-5D is being used in two quality indicators showing the proportion of patients with knee and hip osteoarthritis who reach the target level for improved HRQoL 1 year after participation in an osteoarthritis learning programme [43]. An improvement in HRQoL is defined as an absolute increase of 0.1 on the EQ-5D index. The goal is for 30% of the patients to reach this level 1 year after participating in the learning programme.
The Swedish Hip Arthroplasty Register (SHPR)	EQ-5D is being used in the quality indicator “Patient-reported results of total hip arthroplasty” [44]. Previously, the indicator was based on the relationship between the patients’ expected and observed EQ-5D-3L index values 1 year after surgery (Observed results/Expected results × 100). The expected EQ-5D index value was calculated based on the patients’ age, sex, comorbidities, diagnosis, and the EQ-5D measurement before surgery. A value over 100 indicated that the observed result was better than the expected. In 2019, the EQ-5D index value in this indicator was replaced by EQ VAS. EQ-5D results are also reported to the SVEUS platform.
The Swedish Spine Register (SWESPINE)	Absolute and case-mix adjusted EQ-5D index values 1 year after surgery are presented as quality indicators on the SVEUS platform [45].
**Quality indicators based on outcome measures - published by the registries**	
The National Quality Registry for Child and Adolescent Habilitation (HabQ)	The item regarding pain and discomfort in EQ-5D-Y is used in a quality indicator for the follow-up of the habilitation of children with cerebral palsy [46]. The goal is that less than 5 % report severe pain or discomfort at the follow-up point when the children are 9 years old.
The Swedish Neuro Registries – Multiple Sclerosis	The EQ-5D index is used as a quality indicator in the quarterly reports that are sent to the contributing units. The quality indicator represents the proportion of patients in each region with an index value < 0 (defined as worst imaginable), 0–0.5 (poor health), 0.5 < 1 (well, not full health), and 1 (full health).
The National Quality Registry for Dependency (SBR)	The average EQ VAS results are used as a quality indicator in the monthly or quarterly reports to contributing units.
The Swedish Hip Arthroplasty Register (SHPR)	The average increase in EQ VAS score, 1 year after surgery, is used as one out of eight quality indicators in a value compass [31].
The Swedish Heart Failure Registry (SwedeHF)	The average EQ VAS outcome for patients with moderate or pronounced impaired systolic function will be used as a quality indicator for health status in a new online tool that is about to be launched. The results of a set of quality indicators are presented for each of the reporting units.
**Quality indicators based on process measures**	
The National Quality Registry for Podiatric Surgery (RiksFot)	The number of EQ-5D-3L measurements is used in a quality indicator in “Health care in numbers”. The quality indicator represents the proportion of patients who have reported their health with EQ-5D and the Self-reported foot and ankle score (SEFAS) before surgery [47].
National Quality Registry for Systemic Psoriasis Treatment (PsoReg)	The number of EQ-5D-3L measurements (descriptive system) from PsoReg is used by the National Board of Health and Welfare in two quality indicators representing the proportion of persons with severe psoriasis who have received a structured assessment of treatment effects with clinical measures and PROM, each year and 3 to 12 months after initiation of systemic treatment [48]. EQ-5D-3L is one of three instruments that should be included in the structured assessment.
National Quality Registry for Congenital Heart Disease (SWEDCON)	The number of EQ-5D-3L measurements are used in a quality indicator aiming to stimulate the follow-up of the national mission to provide surgery for Grown Up Congenital Heart Disease (GUCH) at Sahlgrenska University Hospital and Skåne University Hospital [49]. The quality indicator summarises the proportion of patients who have had a follow-up regarding their quality of life 6–18 months after surgery.

Note: *HRQoL* health-related quality of life, *PROM* Patient-reported outcome measure, *VAS* visual analogue scale

The outcome-based quality indicators are  most commonly based on the EQ-5D index values, or on results from EQ VAS. One of the registries has chosen to focus on one of the items (pain/discomfort) of the child-friendly version EQ-5D-Y as a quality indicator.

#### Use of EQ-5D data in benchmarking

Seven registries reported that their EQ-5D data are used for benchmarking (Table 4). Health care regions, or contributing units, are most commonly compared in terms of EQ-5D index values. In two cases, results from EQ VAS are compared. The comparisons are either presented on the registries’ web pages, in annual reports, in reports to the individual units, or through other portals such as “Health care in numbers” [50] or SVEUS [45]. Adjustments for differences in patient characteristics (case-mix) were only reported in two cases.

**Table 4:** Use of EQ-5D data for benchmarking

Registry	Description of benchmarking
The registry for Better management of patients with OsteoArthritis (BOA)	Quality indicators based on EQ-5D index data from BOA, presented in “Health care in numbers” (Table 3), are not only presented on a national level, but also per region.
The Swedish Hip Arthroplasty Register (SHPR)	The quality indicator based on EQ VAS data from SHPR presented in “Health care in numbers” (Table 3) are presented per reporting unit, and on a regional and national level. Observed outcomes are compared with expected outcomes (based on age, sex, diagnosis, Charnley class and pre-operative PROM results).
Swedish Spine Register (Swespine)	Individual clinics connected to the registry may create reports with their own EQ-5D results compared to those of other clinics and the national average. Absolute and case-mix adjusted EQ-5D index values 1 year after surgery are also presented per unit for those units that are connected to the SVEUS platform [45].
The Swedish Neuro Registries – Multiple Sclerosis	The EQ-5D index is presented per region on the registry’s Visualisation and Analysis Platform (VAP), which can be accessed by health care providers [29]. In the quarterly reports to the units, the results of the quality indicator for each unit are also presented in comparison with the results of other units.
The Swedish Quality Registry for Pain Rehabilitation (SQRP)	The average EQ-5D index and EQ VAS scores, before and after pain rehabilitation, are presented for all contributing units in the annual report [51]. The number of patients with improvements, no change, or deteriorations in the EQ-5D index and EQ VAS are also presented per unit. A change in the EQ-5D index is defined as a difference of more than 0.1 before and after rehabilitation. For EQ VAS, a change is defined as at least a 20% difference between the measurements.
National Quality Registry for Systemic Psoriasis Treatment (PsoReg)	The average EQ-5D index is presented per clinic in the annual report [52].
The National Quality Registry for Podiatric Surgery (RiksFot)	The average EQ-5D index before, and 1 year after, surgery are for all contributing units publicly available on the registry webpage [40].

Note: *PROM* Patient-reported outcome measure, *VAS* visual analogue scale

#### Use of EQ-5D data for quality improvement

Six registries reported that EQ-5D data from their registry have been used for quality improvement (CPUP, MMCUP, BOA, Riksfot, SQRP, the Neuro registry for Motor Neuron Disease). However, quality improvement projects are most commonly initiated locally by the health care providers and the representatives for the registries could in most cases not provide detailed information regarding these initiatives.

#### Use of EQ-5D results during individual patient consultations

EQ-5D data from seven registries were reported as being used for screening and monitoring, as supporting clinical decisions for individual patients, and/or for shared decision-making during individual patient consultations (Table 5). For these purposes, the patients’ individual data are most commonly used, but there is also one example of aggregated data being used to inform how other patients have experienced their HRQoL after surgery (RiksFot).

**Table 5:** Use of EQ-5D results during individual patient consultations (with the purpose of screening, monitoring, decision aids and/or shared decision-making)

Registry	Description of use in decisions at an individual level
The Swedish Neuro Registries – Multiple Sclerosis	The registry provides a clinical support tool, that includes EQ-5D and eleven other health measures, to share the results of each patient with clinicians and the patients themselves. The latest reported results from EQ-5D and eleven other health parameters are presented in the so called Function Watch [29]. In this tool, the individual patient’s outcome is compared to those of a reference group from the registry with comparable characteristics regarding sex, age, MS duration and treatment status of the patient. The tool is used by neurologists to assess whether the outcome of a treatment is acceptable, and what problems should be addressed during the individual patient consultations. The registry has also reported that some neurologists use this information together with the patients when discussing the patient’s situation, and in making decisions regarding treatment.
The Swedish Rheumatology Quality Register (SRQ)	The registry provides care givers and patients with a table overview, including EQ-5D results over time. The overview may be used before and during meetings with the patients for discussion and decisions regarding treatments.
The National MMC Follow-Up Program and Quality of Care Registry (MMCUP)	Results from EQ-5D are used in combination with other measures to secure care for the individual patient, and to support the choice of treatment in the follow-up programme, linked to the National MMC Follow-Up Program and Quality of Care Registry (MMCUP).
The National Quality Registry for Child and Adolescent Habilitation (HabQ)	Individual EQ-5D data are used by the health care providers for individual feedback to patients, and for shared decision-making.
The National Quality Registry for Haemophilia	Individual EQ-5D data are used by the treating units for individual feedback to the patients during consultations, and can be used to monitor the individual patient’s treatment.
The Swedish Neuro Registries – Motor Neuron Disease	Individual EQ-5D results are being used in conjunction with data from the Hospital Anxiety and Depression Scale (HADS) to make decisions about treatment with antidepressants for individual patients. If the EQ-5D scores are low but the depression scores are satisfying, EQ-5D reveals the need to identify other reasons for poor HRQoL.
The National Quality Registry for Podiatric Surgery (RiksFot)	Aggregated EQ-5D data are used to show patients facing surgery how a group of patients who have undergone the corresponding surgery experience their symptoms 1 year later.

## Discussion

### Main findings

This study presents a real-world case where a specific PROM has been implemented on a large scale in routine health care. It intends to contribute to the knowledge regarding routine collection of PROMs in clinical practice by describing how EQ-5D is collected, presented, and used for different purposes. Our findings indicate that EQ-5D is most commonly collected within registries related to the musculoskeletal system, and that it has a wide application among the disease areas covered by the registries. Data collection has increased continuously, with growing interest in EQ-5D-5L over the last 7 years.

Most registries provide some feedback regarding the results of the EQ-5D data collection to patients, clinicians, or other decision-makers, most commonly on an aggregated level. Individual responses are, to some extent, also made available to treating clinicians and/or the individual patients, e.g. for shared decision-making. The collected EQ-5D data are most commonly used for assessing interventions, and as quality indicators for following up the quality of care. The provided examples illustrate how the EQ-5D data can be analysed and summarised to provide information for decisions at different levels of the Swedish health care system.

Yet, there are areas for improvement to consider. Although EQ-5D outcomes are made available and are being used, it is, based on our findings, unclear whether the data influence actual health care decisions, and if such influence leads to better health outcomes. To improve the quality of care, information from quality indicators and benchmarking needs to be actively used in quality improvement efforts, which was reported by only a few of the registries. In addition, representatives from almost 50% of the registries reported that the EQ-5D data are not being used, or that they are unaware of whether the data are being used.

### Interpretation of study findings and comparison to previous research

Limited use of PROMs data has previously been observed in initiatives for implementing PROMs in clinical practice [23]. Proposed explanations include a perception that the collected data are not fit for purpose, and that there are challenges associated with accessing, understanding and acting on the results [19]. Several authors have emphasised the importance of the collected data being perceived as relevant and meaningful by the health care personnel who invest their time and effort to collecting it [5, 20, 21, 53]. Thus, for a successful implementation in clinical practice, it has been recommended to have a clear goal when starting the collection, and to involve relevant stakeholders in the planning phase [21].

The incentives for including PROMs in the NQRs have, until recently, largely focused on whether PROM data are collected. In 2010, the collection of patient-reported measures became a requirement for the registries to reach a certain certification level [9]. As the certification level is linked to the financing of the registry [54], it has likely created an incentive for the registries to primarily initiate the collection of PROM data, which has also been mentioned by several of the registry representatives. Today, data collection from patient-reported measures is still a criterion in the certification process, but only if it is considered relevant to the scope of the registry [54]. More importantly, the revised criterion is focused on whether data from patient-reported measures are made publicly available.

Since 2014, the registries also need to specify how PROM data are used for quality improvement in their annual funding applications submitted to SALAR [9]. However, this is usually a result of local initiatives and, as supported by our findings, the registries may be unaware whether the data are used for quality improvement or not. Notably, several representatives from the registries commented that there is no demand, or only a limited demand, for the EQ-5D data from health care providers. Whether this is because the health care providers do not know that the data are available, or how it can be used, or if they do not find the data relevant, was difficult to assess in this study.

For the results to be actionable, previous literature emphasizes that the data must be analysed and presented in a way that users can easily interpret and understand [24, 55]. Interestingly, our study has shown that the analysis of the EQ-5D data from the registries has quite often been focused on the mean EQ-5D index value, which summarises the responses to the five questions into one overall index, based on country-specific value sets. These value sets are mainly developed for use in health economic evaluations, and are most commonly based on techniques for eliciting preferences for hypothetical health states among the general public [15]. However, our study reveals that the use of the EQ-5D data from the NQRs is considerably focused on the clinical context. For such purposes, alternative analysis methods have been recommended, that place more focus on the patients’ responses to the individual items, and their rating on EQ VAS [56, 57]. Furthermore, local improvement teams may face additional challenges when making use of PROMs data from the NQRs in local quality improvement initiatives, as these may have differing needs regarding the type of instrument, as well as the timing and frequency of measurements.

Other possible explanations for the relatively limited reported use of EQ-5D data from the NQRs are that some of the registries only recently began collecting data, that the coverage is too low for the analysis to be considered meaningful, or that the use of the data is more focused around other measures collected in the registry. Hence, attention must be given to that the NQRs are collecting other health outcome measures, and that the findings and interpretations from our study are limited to the use of EQ-5D data only.

### Strengths and limitations

The information in this study has been collected in collaboration with at least one representative from each of the included registries, and we have therefore had access to the most updated information from each registry. All representatives had the opportunity to confirm or modify the compiled information regarding the collection, presentation, and use of EQ-5D data from the registry.

Yet, relying on information from one or two representatives may also have some limitations. First, the information provided may be influenced by whom we were in contact with. The representatives from the registries may have interpreted the questions differently. Thus, it is possible that some information may have been missed, and our aggregated quantitative analysis should consequently be interpreted with caution. Nevertheless, the findings on the specific uses of EQ-5D data have been strengthened by the fact that we have asked for examples and references.

Second, as previously mentioned, the representatives from the registries may be unaware of how the data have been used in clinical practice, or in decision-making by other stakeholders. This study limitation is especially prominent in the interpretation of use in quality improvement. As quality improvement work is most commonly performed locally by the health care providers, we have not been able to go into depth regarding the characteristics of these initiatives. We found that collecting this information would have required interviews with a large number of care givers, which was beyond the scope of this study. Thus, additional studies are needed to explore how PROMs are currently integrated in quality improvement initiatives in health care.

Third, the template that was distributed and discussed together with the registry representatives included both open questions and examples (e.g., of channels for feedback and use of data). It could be considered a limitation that examples were provided, as this may have influenced their responses. However, the personal communications with the registry representatives enabled good dialogue regarding the questions in the template, and how these were applicable for each particular registry.

Finally, we have only been in contact with the registries that currently have a license for using EQ-5D. Thus, we do not have any information on why other registries have decided not to use EQ-5D, or why some registries that previously collected EQ-5D data have stopped.

### Implications

The initiative to collect EQ-5D within the Swedish NQRs is one of the most ambitious large-scale PROM implementations that we have been able to identify in the literature. Our study is the first to describe this initiative in detail, and contains several valuable examples of how PROM data can be collected and used for follow-up and decision-making. It also shows a potential for improving the use of EQ-5D, such as setting up clear goals for data collection, together with the stakeholders, and considering alternative data analyses in order to improve the interpretability of the results, including a focus on the specific items of EQ-5D, and case-mix adjustments. In this process, the guidelines developed by ISOQOL may provide useful guidance considering aspects such as the purpose of collecting the data, key barriers that need to be addressed, and how, when, and where the results could be presented [18, 21].

Our findings suggest that the case of EQ-5D within the NQRs provides opportunities to study whether the use of PROMs leads to actual improvements in patient care. Although some previous studies indicate that the use of PROMs is associated with improved communication and decision-making [58], reviews of the scientific literature have highlighted that there is limited evidence for the effects from implementing and using PROMs in clinical practice [5, 58–60]. Thus, there is a need for future studies to investigate if the use of PROMs has an actual effect on the decisions made by patients and care givers, and ultimately improves the care and health of the patients. The examples presented in our study may provide useful real-world cases for follow-up.

## Conclusions

This study contributes to the field of routine collection of PROMs in clinical practice by describing how EQ-5D data are collected within the Swedish NQRs, as well as how results are presented and used at different levels of the Swedish health care system. It shows that the collection and use of EQ-5D data vary across the registries, which may be explained in part by the different purposes of the registries. Although our study has shown multiple valuable examples of the usefulness of EQ-5D data as a PROM, it has also revealed that there is potential for improving feedback and the use of the data at different levels of the health care system.

## Supplementary information

**Additional file 1: Table S1.** Template requesting information regarding the registries’ collection, presentation, and use of EQ-5D (originally in Swedish). **Table S2.** Collection of EQ-5D. Registries within the category of Cancer. **Table S3.** Collection of EQ-5D. Registries within the category of Circulatory system. **Table S4.** Collection of EQ-5D. Registries within the category of Endocrine organs. **Table S5.** Collection of EQ-5D. Registries within the category of Infection. **Table S6.** Collection of EQ-5D. Registries within the category of Musculoskeletal system. **Table S7.** Collection of EQ-5D. Registries within the category of Nervous system. **Table S8.** Collection of EQ-5D. Registries within the category of Paediatrics. **Table S9.** Collection of EQ-5D. Registries within the category of Psychiatry. **Table S10.** Collection of EQ-5D. Registries within the category of Stomach and intestines. **Table S11.** Collection of EQ-5D. Registries within the category of Other areas (rare diseases). **Table S12.** Collection of EQ-5D. Registries within the category of Other areas (skin disease).

## Data Availability

The majority of data generated or analyzed during this study are included in this published article [and its supplementary information files]. Any additional data used and/or analyzed during the current study are available from the corresponding author on reasonable request.

## Abbreviations

HRQoL: Health-related quality of life
ISOQOL: International Society for Quality of Life Research
NQRs: National Quality Registries
PROM: Patient-reported outcome measure
SALAR: Swedish Association of Local Authorities and Regions
