# Supplementary material for: Collection and use of EQ-5D for follow-up, decision-making, and quality improvement in health care - the case of the Swedish National Quality Registries
Source: J Patient Rep Outcomes. 2020 Sep 16;4:78. doi: 10.1186/s41687-020-00231-8 (PMC7494720; doi:10.1186/s41687-020-00231-8)
Supplement: Supplementary file 1 — Additional file 1: Table S1. Template requesting information regarding the registries’ collection, presentation, and use of EQ-5D (originally in Swedish). Table S2. Collection of EQ-5D. Registries within the category of Cancer. Table S3. Collection of EQ-5D. Registries within the category of Circulatory system. Table S4. Collection of EQ-5D. Registries within the category of Endocrine organs. Table S5. Collection of EQ-5D. Registries within the category of Infection. Table S6. Collection of EQ-5D. Registries within the category of Musculoskeletal system. Table S7. Collection of EQ-5D. Registries within the category of Nervous system. Table S8. Collection of EQ-5D. Registries within the category of Paediatrics. Table S9. Collection of EQ-5D. Registries within the category of Psychiatry. Table S10. Collection of EQ-5D. Registries within the category of Stomach and intestines. Table S11. Collection of EQ-5D. Registries within the category of Other areas (rare diseases). Table S12. Collection of EQ-5D. Registries within the category of Other areas (skin disease). [file 41687_2020_231_MOESM1_ESM.docx]

**Supplementary material**

**Table S1. Template requesting information regarding the registries’ collection, presentation, and use of EQ-5D (originally in Swedish)**

**Name of registry:**

**Contact person:**

**Date for the information collection:**

| **General information** |
| --- |
| What patients are included in the registry? |
| How is it decided that they will be included in the registry (at a health care visit, at diagnosis, etc.)? |
| When is the last registration made in the registry for each patient (24 months after intervention, when the patient dies, etc.)? |
| What year did the registry start collecting data? |
| How many patients are registered in total, and per year? |
| What is the estimated coverage? How many of all possible clinics in Sweden participate in the collection? How many of all eligible patients are given the opportunity to respond to the questionnaires? What is the response frequency? |
| What background variables are registered for each patient (e.g., sex, age, geographical affiliation, ethnicity, socioeconomics, marital status, education)? |
| What background information is registered regarding the patients’ disease? |
| What diagnostic tests and treatments are registered for the patients? |
| **Collection of PROM data** |
| For what patients are you collecting PROM, and specifically EQ-5D data? |
| Which version of EQ-5D are you using?   - - Which versions (e.g., EQ-5D-3L, EQ-5D-5L)?   - Is EQ VAS included? |
| What value set are you using to calculate the EQ-5D index? |
| What other PROM instruments are you using? |
| How many EQ-5D measurements do you have in total? For how many patients have you registered EQ-5D data, per year? |
| When did you start collecting EQ-5D data (year, month)? |
| Are you measuring once or several times per patient?  When are data collected (e.g., pre-post treatment, at all visits, at follow-up at 3, 6, 9 months, or when patients do their own registration at home)? |
| **Administration of PROM instruments** |
| How is data collected (e.g. through a paper questionnaire, web survey, or face-to-face interviews)? If interview, who is doing the interview? |
| Are the patients responding themselves or are other responders acting as proxies? |
| **Use of PROM data for quality improvement and decision-making** |
| How are PROMs data made available to decision makers (patients, clinicians, directors, politicians)?   - Individual feedback to the patient (e.g., instantaneous or at follow-up) - Individual feedback for shared decision making, or decision support to the patient and clinicians - Aggregated feedback in clinical setting to the patient - Aggregated feedback in clinical setting to the health care professionals - Aggregated feedback, other (e.g., webpage or annual report) - Research (in what publications?)   - R-RCT? (Registry-based randomised controlled trials) |
| How is PROM data analysed and used today?   - Quality improvement (examples)? - Guidelines and/or recommendations? - Benchmarking? - Evaluation of treatment/intervention effects? - Health economic evaluations? |

**Table S2. Collection of EQ-5D. Registries within the category of Cancer**

| Registry (start year) | Patients with EQ-5D data | Start year for collection of EQ-5D data | Version of EQ-5D | Number of EQ-5D measurements /Total number of individuals or cases in the registry | When is EQ-5D data collected? | How is EQ-5D data collected? | Other patient-reported outcomes collected |
| --- | --- | --- | --- | --- | --- | --- | --- |
| National Quality Registry for Oesophageal and Stomach Cancer, NREV (2007) | Patients with a diagnosis of oesophageal and stomach cancer | 2009 | EQ-5D-3L (descriptive system and EQ VAS) | A total of 2420 EQ-5D measurements /Until 2016 the registry contained a total of 6292 cases of oesophageal and 6939 cases of stomach cancer) | Once 1 year after diagnosis | A paper questionnaire is sent home to the patients | EORTC QLQ-C30, QLQ-OG25 |
| National Prostate Cancer Register (NPCR) of Sweden (1998) | Two PROM programs: everyone on radical therapy (e-PROM curative) and patients with advanced prostate cancer (PPC) | 2018 | EQ-5D-5L (descriptive system and EQ VAS) | Approximately 100 EQ-5D measurements /A total of 181 747 persons are included in the registry | E-PROM curative: Before and after treatment and at 1 and 5 years after treatment (Since 2018 also at 3 months after treatment). PPC: Continuously when there is a need. | Web application (INCA) | IIEF, and questions about stress, urinary incontinence, bowel disorders |
| National Quality Registry for Head and Neck Cancer, SweHNCR (2008) | Patients with head and neck cancer | 2018 | EQ-5D-5L (descriptive system and EQ VAS) | The response rate of the PROM questionnaire is ≈ 60% the first months of collection  /A total of 1500 patients are registered per year | Once 4-6 months after diagnosis | A paper questionnaire is sent home to the patients | For oral cavity cancer: EORTC QLQ-C30, QLQ-H&N35, QLQ-FA12 |

Note: EORTC: European Organization for Research and Treatment of Cancer, IIEF: International Index of Erectile Function, QLQ-C30: Quality of Life of Cancer Patients (core), QLQ-FA12: Cancer related fatigue (module), QLQ-H&N35; Head & neck cancer (module), QLQ-OG25: Oesophago-Gastric (module), VAS: Visual Analogue Scale.

**Table S3. Collection of EQ-5D. Registries within the category of Circulatory system**

| Registry (start year) | Patients with EQ-5D data | Start year for collection of EQ-5D data | Version of EQ-5D | Number of EQ-5D measurements /Total number of individuals or cases in the registry | When is EQ-5D data collected? | How is EQ-5D data collected? | Other patient-reported outcomes collected |
| --- | --- | --- | --- | --- | --- | --- | --- |
| National Quality Registry for Atrial Fibrillation and Anticoagulation, AuriculA (2007) | Patients with atrial fibrillation visiting the clinics and participating in the ABCAF-study. Data may also be collected for other patients but there is no routine for this | 2018 | EQ-5D-5L (descriptive system and EQ VAS) | A total of 65-70 measurements (February 2019) /A total of 210 000 unique patients | Baseline with follow-up at 1 and 2 years after baseline | Paper questionnaire (baseline) or web application for the follow-ups | ASTA |
| National Quality Registry for Congenital Heart Disease, SWEDCON (adults 1992, children 1986) | Adults with grown up congenital heart disease (GUCH) | 2009 | EQ-5D-3L (descriptive system and EQ VAS) | A total of 25 241 EQ VAS measurements for 9 872 unique patients and even more for the descriptive system (until 2019-06-27). A total of 3 425 EQ-5D measurements during 2018 (of 5 801 registered health care visits by patients with GUCH). /A total of 56 000 patients in the registry at the end of 2017 (of these approximately 15 000 are adults). | At most health care visits, but it may vary between centres. The ambition is before and after treatment. | Paper questionnaire | DISABKIDS and PedsQL (for children) |
| National Quality Registry for Enhancement and Development of Evidence-Based Care in Heart Disease, Swedeheart (2005) | Patients who are followed in the Secondary Prevention after Heart Intensive Care Admission (SEPHIA) registry | 2005 | EQ-5D-3L (descriptive system and EQ VAS) | A total of 165 743 EQ-5D measurements (including year 2018), 16 291 per year (2018) /A total of approximately 80 000 registered patients (2005-2018) and 7-8 000 patients per year in SEPHIA | At follow-up visit at 6-10 weeks and 12-14 months after myocardial infarction | Some hospitals send the questionnaires home to the patients before a physical or telephone visit, some distribute it in the waiting room before the visit. One hospital uses web application | Haskell’s questions about physical activity and exercise  Frändin/Grimby activity scale.  Diet index based on question about healthy eating habits by the National board of Health and Welfare. |
| The Swedish Catheter Ablation Registry (2005) | Patients with a relevant diagnosis undergoing a catheter ablation for a dysrhythmia | 2017 | EQ-5D-3L (descriptive system and EQ VAS) | A total of 237 EQ-5D measurements (only year 2017) /Approximately 45 000 interventions and 35 000 patients are registered | Before the intervention and follow- up at 1 and 5 years | Varies between different centres, mainly via a paper questionnaire or a web application | Questions regarding inconvenience related to dysrhythmia, and how bothered the patient is in daily living when experiencing dysrhythmia. |
| The Swedish Heart Failure Registry, SwedeHF (2003) | Patients in the registry, which are adult patients with a diagnosis of heart failure | 2005 | EQ-5D-3L (descriptive system and EQ VAS) | A total of 36 817 EQ-5D measurements for 29 756 unique patients /A total of 95 752 unique patients and approximately 9 000 per year | Index with follow-ups within 6 months and 1 year. Thereafter annual measurements. | A questionnaire is sent home to the patients before the care visit and the responses are registered at the visit. Alternatively, the responses are registered during a phone contact. | Patient-reported symptoms related to heart failure: breathlessness, fatigue. |
| The Swedish National Quality Registry for Ulcer Treatment, RiksSår (2009) | In a study of patients diagnosed through E-health (E-hälsa) 2014-2016 | 2014 | EQ-5D-3L (descriptive system and EQ VAS) | A total of 100 patients (until 2016) /8144 patient registrations (including 2017), approximately 1 000 new patients per year | At diagnosis and when wound is healed | Paper questionnaire | KUPP |
| The Swedish Register for Cardiopulmonary Resuscitation, SRCR (1990) | Adult patients who survive a cardiac arrest and are alive after 3 months | 2013 | EQ-5D-5L (descriptive system and EQ VAS) | A total of 2395 EQ-5D measurements (until year 2017) /87 811 patients with cardiac arrest outside of hospital (1990-2016) and 31 971 cardiac arrests at hospitals (2005-2016) | Once 3-6 months after cardiac arrest. | A questionnaire is sent home to the patients together with a time for a phone appointment. During the phone call, an interviewer goes through the questionnaire with the patient | HADS, LiSat-11 (since mid-2018).  Separate questions regarding mental/intellectual recovery, daily activities and occupation. |

Note: ASTA: Arrhythmia-Specific questionnaire in Tachycardia and Arrhythmia, HADS: Hospital Anxiety and Depression Scale, KUPP: Quality from the Patient’s Viewpoint, LiSat-11: Life Satisfaction Questionnaire, PedsQL: Pediatric Quality of Life Inventory, VAS: Visual Analogue Scale.

**Table S4. Collection of EQ-5D. Registries within the category of Endocrine organs**

| Registry (start year) | Patients with EQ-5D data | Start year for collection of EQ-5D data | Version of EQ-5D | Number of EQ-5D measurements /Total number of individuals or cases in the registry | When is EQ-5D data collected? | How is EQ-5D data collected? | Other patient-reported outcomes collected |
| --- | --- | --- | --- | --- | --- | --- | --- |
| National Quality Registry for Pituitary Disease (1991) | Patients with pituitary tumours or pituitary diseases | 2011 | EQ-5D-3L (descriptive system and EQ VAS) | EQ-5D measurements at 1, 5 and 10 years, respectively, by diagnosis (2016):  Patients with acromegaly: 74, 135 and 96 EQ-5D measurements.  Patients with Mb Cushing: 29, 57 and 34 EQ-5D measurements.  Patients with prolactinoma: 137, 213 and 158 EQ-5D measurements.  Patients with with non-hormone producing pituitary tumour (NFPA): 257, 391 and 227 EQ-5D measurements.  /A total of 8 000 patients registered in the registry and approximately 300-500 patients per year (until 2017) | First health care visit and annual follow-ups | Paper questionnaire | Working ability |

Note: VAS: Visual Analogue Scale.

**Table S5. Collection of EQ-5D. Registries within the category of Infection**

| Registry (start year) | Patients with EQ-5D data | Start year for collection of EQ-5D data | Version of EQ-5D | Number of EQ-5D measurements /Total number of individuals or cases in the registry | When is EQ-5D data collected? | How is EQ-5D data collected? | Other patient-reported outcomes collected |
| --- | --- | --- | --- | --- | --- | --- | --- |
| National Quality Registry for Infectious Diseases (2007) | Patients in the registries for bacterial meningitis and endocarditis | 2013 | EQ-5D-5L (descriptive system and EQ VAS) | A total of 125 EQ-5D measurements for bacterial meningitis (including 2018). Unclear for endocarditis as the collection has just started. /In total, 1 173 patients with bacterial meningitis (approximately 250 per year) and 5 013 patients with endocarditis (approximately 600 per year) have been included in the registry. | At discharge and follow-up at 6 months | Paper questionnaire | Follow-up questions regarding bacterial meningitis and endocarditis |
| National Quality Registry for Primary Immunodeficiency, PIDcare (2012) | Voluntary for patients with primary and secondary immunodeficiency disease as part of the Health diary (“Hälsodagboken”). Patients with HIV are not included as they are registered in another registry. Not implemented by all clinics | 2016 | EQ-5D-5L (descriptive system and EQ VAS) | In total, 237 EQ-5D measurements (including 2017) / The total number of patients in the registry (including 2017) is 2 063 (347 included during 2017). | Some complete EQ-5D every week, others only once. Planning to ask the patients to complete EQ-5D and RAND36 at specific time points | Web application | Symptom diary, sick leave, hospitalizations. Will be complemented with RAND-36, more symptoms, and the possibility to register own symptoms |

Note: VAS: Visual Analogue Scale.

**Table S6. Collection of EQ-5D. Registries within the category of Musculoskeletal system**

| Registry (start year) | Patients with EQ-5D data | Start year for collection of EQ-5D data | Version of EQ-5D | Number of EQ-5D measurements /Total number of individuals or cases in the registry | When is EQ-5D data collected? | How is EQ-5D data collected? | Other patient-reported outcomes collected |
| --- | --- | --- | --- | --- | --- | --- | --- |
| Better management of patients with OsteoArthritis, BOA (2008) | Patients with osteoarthritis in hips, knees or hands who are registered for a first visit to receive structured treatment | 2008 | EQ-5D-5L (descriptive system and EQ VAS)  Changed from EQ-5D-3L 2017 | EQ-5D measurements for 97 000 patients in total (until 2018-11-19) and for 15 000 patients per year. A total of 213 400 measurements (including all follow-ups) /106 886 individuals included in the registry (2018-11-19). Approximately 18 300 individuals included during 2017. | At first visit and at follow-ups at 3 and 12 months | Paper questionnaire or web application | Questions about pain. Among others: • How often do have pain in any of your joints?  • Your average pain in your most painful joint the last week? • Are you afraid that your joints will get hurt by physical exercise/activity?  • Do you have that much pain from any of your joints that you would like to have surgery? |
| National Quality Registry for Hip Fracture Patient Care, RIKSHÖFT (1988) | Patients with all types of hip fractures and surgical methods. The reporting hospitals decide on which patients to collect EQ-5D data | 2008 | EQ-5D-5L (descriptive system and EQ VAS)  Changed from EQ-5D-3L 2017 | A total of 37 886 EQ-5D measurements (including 2017), approximately 2 000 measurements per year/ More than 300 000 registered patients, 13 272 registered patients during 2017 | The hospitals decide whether to register once or twice per patient. At hospitalization, patients are asked how it was the week before fracture. Follow-up at 4 months after surgery | Paper questionnaire or telephone interview | Questions on pain from the hip, if the patient is taking analgesics due to pain in the hip, walking functionality. Other PROMs (e.g. FES and PRP) have been used in specific studies |
| The National Quality Registry for Podiatric Surgery (RiksFot) | Patients who have received elective foot surgery at a unit reporting to the register. | 2015 | EQ-5D-3L (descriptive system and EQ VAS) | In total, approximately 11 500 EQ-5D measurements (pre- and post-surgery) /In total, 11 600 operations are registered (2019-01-25). The number of registered operations increase each year (3 753 in 2017) | Before and after intervention (1 year after surgery) | Paper questionnaire preoperatively, paper questionnaire or web application (link in e-mail) postoperatively | SEFAS, degree of satisfaction of the surgery |
| The Swedish amputation and prosthesis register, SwedeAmp (2011) | Patients with a lower limb amputation (transtibial or higher level) | 2011 | EQ-5D-5L (descriptive system and since 2018 also EQ VAS)  Changed from EQ-5D-3L 2017 | EQ-5D-3L (including 2017): 472 at 6 months, 349 at 12 months, 157 at 24 months.  EQ-5D-5L (including 2018): 394 at 6 months, 286 at 12 months, 177 at 24 months) /A total of 5 762 patients and 7 776 interventions in the registry (including 2018) | Follow-ups at 6, 12 and 24 months after amputation | Paper questionnaire during health care visit or during telephone interview | General health, LCI-5, Phantom limb and stump pain, Prosthetic Use score, Socket comfort score, capability to take on/off the prosthesis, use of assistive devices and wheelchair |
| The Swedish Ankle Registry, Swedankle (1997) | Patients who have had a primary intervention with total ankle prosthesis, prosthetic revisions, ankle arthrodesis or angular corrective surgery | 2008 | EQ-5D-3L (descriptive system and EQ VAS) | Approximately 5 000 EQ-5D measurements (2019-01-03) /Approximately 4 000 patients in the registry and 400 new cases registered each year | Before and after intervention (follow-up at 6, 12 and 24 months) | Paper questionnaire at health care visit preoperatively, paper questionnaire by mail postoperatively | SF-36, SEFAS, degree of satisfaction of the surgery |
| The Swedish Elbow Arthroplasty Register and The Swedish Shoulder Arthroplasty Register (1999) | Patients undergoing shoulder arthroplasty | 2004 | EQ-5D-5L (descriptive system)  Changed from EQ-5D-3L 2017 | A total of approximately 4 000 (pre-operatively), 3 100 (1 year), 6 500 (5 years) and 1 900 (10 years) EQ-5D measurements /Approximately 2 000 shoulder operations are registered each year | Before intervention and follow-up at 1, 5 and 10 year | Paper questionnaire | WOOS (shoulder arthroplasty), quick-DASH (elbow arthroplasty) and WOSI (shoulder instability) |
| The Swedish Fracture Register, SFR (2011) | Adults with orthopaedic fractures | 2011 | EQ-5D-3L (descriptive system and EQ VAS) | Approximately 180 000 EQ-5D measurements (including injuries dated December 2018) /A total of 375 000 registered fractures (2019-06-10) and approximately 75 000 new cases per year | Before the injury (retrospectively) and 1 year after | Paper questionnaire until February 2019. Awaiting web application. | SMFA |
| The Swedish Hip Arthroplasty Register (1979) | Patients undergoing hip arthroplasty | 2008 | EQ-5D-5L (descriptive system and EQ VAS)  Changed from EQ-5D-3L 2017 | In total, 440 434 EQ-5D measurements (2018-09-13), 11 448 preoperative and 30 210 postoperative EQ-5D measurements during 2017 /More than 360 000 primary operations registered. Approximately 25 000 operations registered per year. | Before intervention and follow-up at 1, 6 and 10 year after surgery | Web application (by link in e-mail) since 2017. For patients without a registered e-mail address, a paper questionnaire by mail | Charnley, hip pain, satisfaction with surgery |
| The Swedish Knee Arthroplasty Register (1975) | Patients who have had a knee prosthesis inserted, removed or replaced at a clinic which has chosen to collect PROMs | 2008 | EQ-5D-3L (descriptive system and EQ VAS) | In 2017, PROM was collected in 37% of the primary operations and 15% of the revisions. /A total of 270 159 primary knee prostheses (since 1975), 14 957 knee prostheses during 2017. | Before intervention and follow-up at 1 year after surgery | Paper questionnaire | KOOS, knee pain, expectations, Charnley, satisfaction with the surgery |
| The Swedish Rheumatology Quality Register, SRQ (1995) | Patients treated for chronic rheumatic diseases | 2008 | EQ-5D-3L (descriptive system) | A total of approximately 300 000 EQ-5D measurements (2018-09-25), and 44 884 per year (2017) /A total of approximately 80 000 patients are included in the registry, 6 804 new patients were included 2017. | At every registration in the patients’ own registration (PER). These are made at health care contacts, visits or telephone contacts, approximately once per year. | Web application at home or at clinic but also possible to use paper questionnaire in connection with the health care visit | Rheumatoid Arthritis and all other rheumatic conditions: Pain (VAS), fatigue due to rheumatic disease, general health due to rheumatic disease, HAQ, swollen and sore joints, symptoms from feet, ability to work.  Specifically for inflammatory back disease: BASFI, BASDAI, BASG  Specifically for inflammatory muscle disease: MAP, SF-36/RAND-36.  Specifically for systemic sclerosis: obstacles in daily activities |
| The Swedish Spine Register, SWESPINE (1998) | All back surgery involving all diagnostic groups (since 2016 back fractures are registered only in the fracture register) | 1998 | EQ-5D-3L (descriptive system and EQ VAS) | In total, 282 673 EQ-5D measurements for spondylosis in the low back and 24 472 for spondylosis in the spine /A total of more than 130 000 registered index operations. Approximately 10 000 operations each year (since 2013) | Before intervention and follow-up at 1, 2, 5, 10 year after the index surgery (reoperations are not followed up) | Paper questionnaire (per mail) and web application (currently only for follow-up) | NDI, ODI, NPRS, Myelopathy scale, SRS-22r, satisfaction with the surgery, Global Assessment; How is your back/leg pain today compared with before surgery? SF-36 has been used but is excluded since 2018. |

Note: BASDAI: Bath Ankylosing Spondylitis Disease Activity Index, BASG: Bath Ankylosing Spondylitis Global assessment, BASFI: Bath Ankylosing Spondylitis Functional Index, DASH: Disabilities of the Arm, Shoulder and Hand, FES: Falls Efficacy Scale, HAQ: Health Assessment Questionnaire, KOOS: The Knee injury and Osteoarthritis Outcome Score, LCI-5: Locomotor Capabilities Index-5, MAP: Myositis Activities Profile, NDI: Neck Disability Index, NPRS: Numeric Pain Rating Scale, ODI: Oswestry Disability Index, PRP: Post Operative Recovery Profile, SEFAS: Self-Reported Foot and Ankle Score, SF-36: The 36-item Short Form Health Survey, SMFA: Short Musculoskeletal Function Assessment, SRS-22r: Scoliosis Research Society Outcomes Questionnaire, VAS: Visual Analogue Scale, WOOS: Western Ontario Osteoarthritis of the Shoulder Index, WOSI: Western Ontario Shoulder Instability Index.

**Table S7. Collection of EQ-5D. Registries within the category of Nervous system**

| Registry (start year) | Patients with EQ-5D data | Start year for collection of EQ-5D data | Version of EQ-5D | Number of EQ-5D measurements /Total number of individuals or cases in the registry | When is EQ-5D data collected? | How is EQ-5D data collected? | Other patient-reported outcomes collected |
| --- | --- | --- | --- | --- | --- | --- | --- |
| Myelomeningocele Follow-Up Programme, MMCUP (2007) | Adults with myelomeningocele (MMC) | 2016 | EQ-5D-5L (descriptive system and EQ VAS) | A total of 102 EQ-5D measurements, and 23 EQ-5D measurements during 2018 (until 4 nov) /A total of 581 patients are included in the registry (nov 2018) and 100 new patients are registered per year. | Annually | Paper questionnaire at health care visit, web-recorded by health care professionals | Questions about pain (inspired by SF-36) for adults. CHQ for children. |
| The National Quality Registry for Rehabilitation Medicine Webrehab Sweden, (1998) | Patients coming to rehabilitation, except those registered in SQRP (see next row) | 1998 | EQ-5D-3L (descriptive system and EQ VAS) | The number of EQ-5D measurements should be the same as the total number of registrations in the registry /A total of 28 378 unique patients are included in the registry. | Before rehabilitation, at the end of the rehabilitation and follow-up at 1 year after ending rehabilitation | Varies between clinics (paper questionnaire, web application, interview) | IPA, LISAT, the patients’ experience of the rehabilitation |
| The Swedish Quality Registry for Pain Rehabilitation, SQRP (1998) | Patients with long-term pain (duration of more than 3 months) | 2009 | EQ-5D-3L (descriptive system and EQ VAS) | A total of 47 000 EQ-5D measurements, 18 000 at the end of rehabilitation /A total of 13 540 patients are registered in the registry (March 2018). | Before rehabilitation, at the end of rehabilitation and follow-up at one year after ending rehabilitation | Paper questionnaire | NPRS, HADS, PCS, WAI.  NRS (specialist care): MPI-s, RAND-36, ISI, Questions about physical activity.  NRS (specialist care), optional: LISAT-11, CPAQ8, Tampa Scale for Kinesiophobia, KASAM, SMBQ, SCI.  NRS (primary care): 2 items in LISAT-11, CPAQ8, FRI, GODIN, PEI  Questions about patient satisfaction (How the rehabilitation has changed the experience of pain and the ability to handle the general life situation) |
| The Swedish Neuro Registries – Hydrocephalus | Adult patients with hydrocephalus | 2016 | EQ-5D-5L (descriptive system and EQ VAS) | EQ-5D measurements before surgery: 85 (2016), 161 (2017) /A total of 5 794 patients are included in the registry (2018-08-31). | Before surgery and follow-up at 3 months, 12 months, 2 years, 5 years and 10 years after surgery | Paper questionnaire or web application | Walking ability, balance, incontinence, memory and need of assistance |
| The Swedish Neuro Registries – Motor Neuron Disease | Patients with Motor Neuron Disease may respond to EQ-5D but it is mainly patients included in the ALSrisc study (patients with ALS who are receiving care at the Karolinska Hospital) who are followed. | 2015 | EQ-5D-3L (descriptive system and EQ VAS) | A total of 616 EQ-5D measurements (including 2018) and approximately 200-300 EQ-5D measurements per year /A total of 871 patients are included in the registry (475 active patients) | Every 6 months | Paper questionnaire or web application (Patient’s own registration, PER) | LISAT, HADS, ADI-12 |
| The Swedish Neuro Registries – Multiple Sclerosis | Patients with multiple sclerosis | 2011 | EQ-5D-3L (descriptive system and EQ VAS) | A total of 32 400 EQ-5D measurements (2019-03-06). In 2018, 7 530 EQ-5D measurements were registered for 1 374 patients /A total of 20 200 patients (2019-03-06) are included in the registry. Approximately 1 000-1 100 patients are registered annually. | In conjunction with every health care visit | Web application (Patients own registration, PER, from home or through web application at the clinic) | MSIS-29, FSMC, fatigue scales, MS symptoms (MS-kollen)  2 domains (fatigue and arm and hand function) of Neuro-QoL have been implemented during 2018. |
| The Swedish Neuro Registries Myasthenia Gravis | Patients with myasthenia gravis. Has not been implemented by all clinicians | 2016 | EQ-5D-3L (descriptive system and EQ VAS) | A total of 59 EQ-5D measurements /A total of 854 patients are included in the registry | Annually | Paper questionnaire | MG-QoL, MG_ADL |
| The Swedish Neuro Registries – Narcolepsy | Patients with Narcolepsy (the goal is that at least half of all counties should register PROMs) | 2017 | EQ-5D-3L (descriptive system and EQ VAS) | Sept 2017: EQ-5D measurements on 19 patients. Aug 2018: 88 EQ-5D measurements on 53 patients /A total of 663 patients are included in the registry (156 new registered patients during 2017) | Every 6 months | Web application (Patients own registration, PER, from home or through web application at the clinic) | ESS, RAND-36, SDQ P, SDQ S |
| The Swedish Neuro Registries – Parkinson’s Disease (2014) | Patients with Parkinson’s disease | 2014 | EQ-5D-5L (descriptive system and EQ VAS)  Changed to EQ-5D-5L in 2018 | A total of 1 944 EQ-5D measurements /A total of 6 432 patients (2019-04-15) are included in the registry, an increase of almost 50 % during the last 2 years | At very health care visit (at least once per year) | Paper questionnaire or web application (Patient’s own registration, PER) | PRO-PD, PDQ-8, UPDRS, NMSQ, ON-OFF (variation of motor symptoms over time) |

Note: ADI-12: ALS Depression Inventory, CHQ: Child Health Questionnaire, CPAQ8: Chronic Pain Acceptance Questionnaire, ESS: Epworth Sleepiness Scale, FRI: Functional Rating Index, FSMC: Fatigue scale for Motor and Cognitive Functions, GODIN: Godin Leisure-Time Exercise Questionnaire, HADS: Hospital Anxiety and Depression Scale, IPA: Impact on Participation and Autonomy questionnaire, ISI: Insomnia Severity Index, KASAM: Sense of Coherence (in English), LISAT: Life Satisfaction Questionnaire, MG_ADL: the Myasthenia Gravis-specific Activities of Daily Living scale, MG-QoL: Myasthenia Gravis Quality of Life, MPI-s: Multidimensional Pain Inventory, MSIS-29: Multiple Sclerosis Impact Scale-29, Neuro-QoL: Quality of Life in Neurological Disorders, NMSQ: Non-Motor Symptoms Questionnaire, NPRS: Numeric Pain Rating Scale, PCS: Pain Catastrophizing Scale, PDQ-8: The Parkinson’s Disease Questionnaire-8, PEI: Patient Enablement Instrument, PRO-PD: The Patient Reported Outcomes in Parkinson's Disease, SF-36: The 36-item Short Form Health Survey, SCI: Stress and Crises Inventory, SDQ: Strengths and Difficulties Questionnaire, SMBQ: Shirom-Melamed Burnout Questionnaire, TSQ: Treatment Satisfaction Questionnaire, UPDRS: Unified Parkinson’s Disease Rating Scale, VAS: Visual Analogue Scale, WAI: Work Ability Index.

**Table S8. Collection of EQ-5D. Registries within the category of Paediatrics**

| Registry (start year) | Patients with EQ-5D data | Start year for collection of EQ-5D data | Version of EQ-5D | Number of EQ-5D measurements /Total number of individuals or cases in the registry | When is EQ-5D data collected? | How is EQ-5D data collected? | Other patient-reported outcomes collected |
| --- | --- | --- | --- | --- | --- | --- | --- |
| National Quality Registry for Child and Adolescent Habilitation, HabQ (HabQ CP: 2005-2018, HabQ autism 2011, HabQ Parental support 2013) | HabQ CP: Children with cerebral palsy  HabQ autism: Children with autism 6 years or older  HabQ Parental support: Children in the follow-up of parental support | 2005 (HabQ CP), 2011 (HabQ autism), 2013 (HabQ Parental support) | EQ-5D-Y (descriptive system and EQ VAS) | HabQ CP: A total of 1237 EQ-5D-Y measurements (proxy 776, self-assessment 461) 2005-2018  HabQ autism: A total of 271 EQ-5D-Y measurements 2011-2017 (until 2017-03-01)  HabQ parental support: A total of 1298 EQ-5D-Y measurements (proxy 1021, self-assessment 277) 2013-2018  /Total number of registered children in the registry:  HabQ CP: 685  HabQ autism: 1624  HabQ parental support: 5118 | HabQ CP, autism and parental support: Proxy responses by parents at specific ages (6, 9, 12, 15, 18 years) and self-assessment at specific ages (9, 12, 15, 18 years)  Since 2018 EQ-5D-Y is only used within HabQ parental support and there is no longer a follow-up at age 12 | Paper questionnaire administrated by curator in interview or sent by mail | HabQ parental support, obligatory part in basic program: SEPS  HabQ parental support, obligatory part in enhanced program: NCSQ, SEPS  HabQ parental support, voluntary part in enhanced program: SSF, HADS, HSQ |
| National Quality Registry for Follow-up of Persons with Cerebral Palsy, CPUP (2005) | Adults with cerebral pares (CP) and who understands the questions. Plan to change to the Swedish 5L version for children when available | 2011 | EQ-5D-5L (descriptive system and EQ VAS)  Changed from EQ-5D-3L 2017 | A total of 1195 EQ-5D measurements.  EQ-5D-5L: 497 persons EQ-5D-3L: 696 persons  /A total of 5 336 persons are registered in CPUP (including 2017). In 2017, 649 newly registered persons (289 adults, 360 children). | Every 2nd or 3rd year (every 3rd year for persons in GMFCS I, every 2^nd^ year for persons in GMFCS II-V). | Paper questionnaire | SF-36 pain, FSS, FMS, Short FES-I |

Note: FMS: Functional Mobility Scale, FSS: Fatigue Severity Scale, GMFCS: Gross Motor Function Classification System, HADS: Hospital Anxiety and Depression Scale, HSQ: Home Situations Questionnare, NCSQ: Nijmegen Child-Rearing Questionnaire (Global skattning av föräldraskap), SEPS: Samhällsstöd och socioemotionellt och praktiskt stöd [In English: Community support and socio-emotional and practical support], SF-36: The 36-item Short Form Health Survey, Short FES-I: Short Falls Efficacy Scale-International, SSF: Styrkor och Stress i föräldraskapet [In English: Strengths and Stress in Parenthood], VAS: Visual Analogue Scale

**Table S9. Collection of EQ-5D. Registries within the category of Psychiatry**

| Registry (start year) | Patients with EQ-5D data | Start year for collection of EQ-5D data | Version of EQ-5D | Number of EQ-5D measurements /Total number of individuals or cases in the registry | When is EQ-5D data collected? | How is EQ-5D data collected? | Other patient-reported outcomes collected |
| --- | --- | --- | --- | --- | --- | --- | --- |
| BipoläR – the Swedish National Quality Register for Bipolar Disorder (2004) | Persons with bipolar affective disorder | 2014 | EQ-5D-3L (descriptive system and EQ VAS) | In total, 19 000 EQ-5D measurements (yearly response rate for EQ-5D of 50%) /A total of 22 050 patients in the registry (including 2017). During 2017, 1 348 new patients were registered (and 4 758 follow-up registrations). | At every outpatient health care visit, approximately once per year. | Interview conducted by nurse or physician | Sleep quality |
| National Quality Registry for Dependency, (2009) | Patients treated in specialised substance abuse and dependency care in county healthcare institutions | 2016 | EQ-5D-3L (descriptive system and EQ VAS) | In total, 14 938 EQ-5D measurements (including 2017). 3 519 unique patients have an EQ-5D measurement during 2017. /The total number of unique patients in the registry is 24 534. During 2017, 7 848 individuals were registered. | At new health care visits and follow-up at 3 and 12 months (significant drop-out at follow-up). | Web application (Region Stockholm) or paper questionnaire | None |
| The Swedish National Quality Register for ECT (2011) | Patients who have been treated with ECT or rTMS | 2011 | EQ-5D-3L (descriptive system and EQ VAS) | In total, 10 630 and 8 534 EQ-5D measurements before and after frequent ECT (including 2017). 1 307 EQ-5D measurements after infrequent ECT. 1 802 EQ-5D measurements at 6 months follow-up. /In total 15 000 patients have been registered in the registry (including 2016). During 2017, 3 427 patients were reported to the registry. | Before and after ECT/rTMS (one week before and within a week after the last ECT/rTMS) and follow-up at 6 months. | Paper questionnaire | MADRS-S, CPRS-memory item, and questions about whether the patients think the ECT/rtMS has helped them |

Note: CPRS: Comprehensive Psychopathological Rating Scale, ECT: Electroconvulsive Therapy, MADRS: Montgomery-Åsberg Depression Rating Scale, rTMS: Repetitive Transcranial Magnetic Stimulation, VAS: Visual Analogue Scale

**Table S10. Collection of EQ-5D. Registries within the category of Stomach and intestines**

| Registry (start year) | Patients with EQ-5D data | Start year for collection of EQ-5D data | Version of EQ-5D | Number of EQ-5D measurements /Total number of individuals or cases in the registry | When is EQ-5D data collected? | How is EQ-5D data collected? | Other patient-reported outcomes collected |
| --- | --- | --- | --- | --- | --- | --- | --- |
| SWIBREG - Swedish Inflammatory Bowel Disease Registry (2005) | Patients with Crohn's disease (CD), ulcerative colitis (UC), the IBD-U (IBD-unclassified) mixed form and microscopic colitis. | 2012 | EQ-5D-5L (descriptive system and EQ VAS) | Approximately 58 800 EQ-5D measurements (including 2018).10 282 EQ-5D measurements during 2018. /44 080 registered patients (August 2018), of which 1 075 are children. | At all health care visits/contacts. | Paper questionnaire or web application | SHS, Symptom scoring |

Note: SHS: Short Health Scale, VAS: Visual Analogue Scale

**Table S11. Collection of EQ-5D. Registries within the category of Other areas (rare diseases)**

| Registry (start year) | Patients with EQ-5D data | Start year for collection of EQ-5D data | Version of EQ-5D | Number of EQ-5D measurements /Total number of individuals or cases in the registry | When is EQ-5D data collected? | How is EQ-5D data collected? | Other patient-reported outcomes collected |
| --- | --- | --- | --- | --- | --- | --- | --- |
| National Quality Registry for Haemophilia (2012) | Patients 15 years or older with haemophilia (the most common are haemophilia A, B and Von Willebrand's disease). | 2017 | EQ-5D-3L and EQ-5D-Y (descriptive system and EQ VAS) | A total of 765 EQ-5D measurements for 469 active patients. 319 EQ-5D measurements for 294 active patients during 2018. /A total of 1 124 registered patients in the register (2019-03-06) | Annually | Paper questionnaire | Chronic pain (yes/no) |

Note: VAS: Visual Analogue Scale

**Table S12. Collection of EQ-5D. Registries within the category of Other areas (skin disease)**

| Registry (start year) | Patients with EQ-5D data | Start year for collection of EQ-5D data | Version of EQ-5D | Number of EQ-5D measurements /Total number of individuals or cases in the registry | When is EQ-5D data collected? | How is EQ-5D data collected? | Other patient-reported outcomes collected |
| --- | --- | --- | --- | --- | --- | --- | --- |
| National Quality Registry for Systemic Psoriasis Treatment, PsoReg (2006) | Patients with psoriasis who receive systemic treatment | 2006 | EQ-5D-3L (descriptive system) | 26 000 EQ-5D measurements for 6 046 patients (until June 2017). 1 165 new EQ-5D measurements for 1 075 patients until June 2017.  /A total of 6 614 patients were registered in 2017 (an increase with 620 patients from 2016). | At every health care contact, approximately 1-4 times per year (not obligatory to use EQ-5D at every visit) | Web application | DLQI |

Note: DLQI: Dermatology Quality of Life Index
